# Machine Learning and SHAP Feature Analysis: Classification Model for Aroma Components in Green Plum Wine

**DOI:** 10.3390/foods15081342

**Published:** 2026-04-13

**Authors:** Xuhui Zhang, Mengsheng Deng, Yu Lei, Yingmei Tao, Shuang Li, Rui Huang, Zonghua Ao, Qiuyun Mao, Xingyong Zhang, Xue Wang, Siyuan Liu, Bingxin Kuang, Chuan Song, Dong Li

**Affiliations:** 1 School of Food and Liquor Engineering, Sichuan University of Science and Engineering, Yibin 644000, China; 2 Luzhou Laojiao Co., Ltd., Luzhou 646000, China

**Keywords:** green plum wine, volatile substances, machine learning, SHAP analysis

## Abstract

This study systematically investigated differences in volatile flavor profiles among fermented green plum wines by integrating gas chromatography–mass spectrometry (GC–MS), sensory evaluation, and odor activity value (OAV) analysis with machine learning and SHapley Additive exPlanations (SHAP) based feature interpretation. The primary objective was to evaluate the applicability of machine learning algorithms for flavor profiling of green plum wine. The results indicated that floral and fruity aromas were predominant in samples NG9, YM7, and YM9. Most green plum wines contained high levels of esters, with ethyl benzoate (up to 4820.53 μg/L), ethyl octanoate (up to 2640.83 μg/L), and benzenecarbaldehyde (up to 3432.96 μg/L) being the major contributors. Among the six classification algorithms compared, fuzzy c-means clustering provided the most distinct clustering structure, identifying three distinct flavor categories. Six machine learning models were subsequently established, of which the decision tree (DT) model exhibited the highest performance, with an accuracy of 95.13%. SHAP analysis further revealed that ethyl octanoate, benzyl ethanoate, and 2-phenylethyl ethanoate exerted the greatest influence on model predictions. Overall, these findings highlight the effectiveness of machine learning as a robust tool for the classification and interpretation of flavor characteristics in fermented fruit wines, with broad applicability in flavor science.

## 1. Introduction

Flavor is a key determinant of fruit wine quality and market competitiveness, being influenced by raw material characteristics, fermentation conditions, and storage processes, all of which directly affect consumer sensory perception [1,2]. Among these attributes, aroma plays a predominant role, arising from both intrinsic fruit constituents and metabolites generated during fermentation. Accordingly, the overall sensory quality of fruit wine is closely linked to its aromatic profile [3]. Green plum is rich in organic acids, aroma precursors, and polyphenolic compounds. During fermentation, these components interact with yeast-derived metabolites, leading to the formation of a diverse range of volatile compounds, including alcohols, esters, aldehydes, and ketones, which collectively contribute to a complex and distinctive aroma profile [4]. High-quality flavor compounds not only enhance the sensory properties of green plum wine but also serve as critical indicators for differentiating product styles and defining quality grades [5]. However, despite its growing popularity, systematic investigations into the aroma composition and objective classification of green plum wine remain limited, particularly in comparison with extensively studied products such as grape wine and Baijiu.

Gas chromatography–mass spectrometry (GC–MS) is one of the most widely applied analytical techniques for aroma characterization [6]. It enables efficient separation and accurate identification of volatile compounds in complex matrices and has been extensively utilized in flavoromics studies [7]. For instance, Huang et al. [8] employed HS-SPME–GC–MS to analyze volatile compounds in various fruit wines, identifying major classes such as esters, alcohols, acids, aldehydes, and ketones. However, with increasing consumer demand for flavor diversity and product consistency, rapid and objective flavor classification remains a major challenge [9]. Traditional sensory evaluation and single-indicator chemical analyses are often subjective, exhibit limited reproducibility, and are inadequate for handling high-dimensional datasets [10].

Recent advances in artificial intelligence (AI) have facilitated the application of machine learning (ML) in food flavor analysis [11]. ML algorithms have demonstrated considerable potential in aroma compound identification, sensory attribute prediction, and quality evaluation [12]. For example, Aliya et al. [13] and Shao et al. [14] successfully applied ML-based models for flavor classification in Baijiu, demonstrating their effectiveness in distinguishing samples with different quality levels. As ML models become increasingly sophisticated, explainable artificial intelligence (XAI) approaches have become essential for enhancing model transparency and reliability [15]. Among these, SHapley Additive exPlanations (SHAP) has been widely adopted to quantify the contribution of individual features to model outputs, providing both global and local interpretability [16,17]. Previous studies have confirmed its effectiveness in identifying key flavor-related variables and improving model interpretability in food systems [18,19], as well as revealing feature interactions and enhancing the consistency of model explanations [20]. However, its application in the aroma analysis and classification of green plum wine remains largely unexplored. Therefore, integrating ML with SHAP-based interpretation offers a promising strategy for systematic flavor characterization and objective classification of fruit wines.

This study focuses on fermented green plum wine and aims to establish a systematic and interpretable framework for flavor evaluation. HS-SPME–GC–MS was employed to characterize volatile compounds, combined with odor activity value (OAV) analysis to identify key aroma-active compounds. Multiple ML models were developed and compared to determine the optimal classification model. Furthermore, SHAP analysis was applied to interpret model predictions and identify the most influential aroma compounds and their contributions to flavor classification. This study provides new insights into the objective classification of green plum wine and proposes an integrated approach combining machine learning with flavor science.

## 2. Materials and Methods

### 2.1. Sample Collection

Green plum raw materials used in this study were sourced from major production regions in Sichuan, Yunnan, and Fujian Provinces, China. Nine representative cultivars were selected, namely Da Bai Mei (DB), Nan Gao Mei (NG), Xing Mei (XM), Yan Mei (YM), Ying Su Mei (YS), Huang Mei (HM), Eryuan Zhaoshui Mei (EZS), Lijiang Zhaoshui Mei (LZS), and Mabian Hong Mei (MBHM). Fruits were harvested at two maturity stages (70% and 90%) based on standardized criteria, including appearance and peel color. In total, 18 representative batches were collected (Table 1). Fresh green plums (5 kg per cultivar) were transported to the laboratory within 48 h of harvest. The fruits were pitted and pulped, followed by enzymatic hydrolysis with pectinase (Nanning Pangbo Bio-Engineering Co., Ltd., Nanning, China) for 30 min. The pH was adjusted to 3.5, and the total soluble solids content was standardized to 20 °Brix. After antimicrobial treatment, Angel BV818 yeast (Angel Yeast Co., Ltd., Yichang, China) was inoculated. Fermentation was carried out at 25 °C for 10 days, after which the wine was clarified and filtered. All samples were processed under identical conditions and stored at 4 °C prior to subsequent analysis of aroma compounds and sensory properties.

**Table 1:** List of abbreviations for different varieties of green plum wine.

Cultivar	Abbreviated Name (70% Maturity)	Abbreviated Name (90% Maturity)
Da Bai Mei	DB7	DB9
Nan Gao Mei	NG7	NG9
Xing Mei	XM7	XM9
Yan Mei	YM7	YM9
Ying Su Mei	YS7	YS9
Huang Mei	HM7	HM9
Eryuan Zhaoshui Mei	EZS7	EZS9
Lijiang Zhaoshui Mei	LZS7	LZS9
Mabian Hong Mei	MBHM7	MBHM9

### 2.2. Sensory Quantitative Descriptive AnalysisMethods

Sensory evaluation was conducted by a trained panel comprising eight assessors (four males and four females, aged 20–36 years). All assessors passed basic taste and olfactory recognition tests and underwent 12 h of systematic training. The training program included the identification of typical wine aroma attributes, calibration of intensity scales, standardization of descriptive terminology, and repeated scoring exercises to ensure the consistency and reliability of the evaluations. Six sensory attributes—floral and fruity aroma, acidity, sweetness, mouthfeel, balance, and overall acceptability—were assessed. Each attribute was rated using a six-point intensity scale (0 = extremely weak; 5 = extremely strong), following a structured sensory evaluation protocol commonly employed in sensory analysis studies. All evaluations were performed in a standardized sensory laboratory under controlled environmental conditions. Samples were coded with random three-digit numbers and evaluated using a single-blind design. The presentation order was randomized. Each sample (10 mL) was served at 20 °C. A 2 min interval was maintained between samples, during which panelists rinsed their mouths with water to minimize sensory fatigue [21].

### 2.3. HS-SPME–GC–MS Analytical Methods

Volatile compounds in fermented green plum wine were analyzed using headspace solid-phase microextraction coupled with gas chromatography–mass spectrometry (HS-SPME–GC–MS). A DVB/CAR/PDMS fiber (50/30 μm; Supelco, 57328-U, Bellefonte, PA, USA) was employed for extraction. Prior to analysis, the fiber was conditioned in the GC injection port at 250 °C for 30 min to remove potential contaminants and ensure extraction stability. All analyses were performed using an Agilent 7890B gas chromatography–mass spectrometry system (Agilent Technologies, Santa Clara, CA, USA). Chromatographic separation was achieved on a DB-WAX polar capillary column (60 m × 0.25 mm i.d., 0.25 μm film thickness; Agilent Technologies). High-purity helium (>99.999%) (Messer Gases Products (Zigong) Co., Ltd., Zigong, China) was used as the carrier gas at a constant flow rate of 1.0 mL/min. The injector temperature was maintained at 250 °C, and injections were conducted in splitless mode. The oven temperature program was as follows: initial temperature of 40 °C (held for 2 min), increased at 5 °C/min to 120 °C (held for 2 min), followed by a ramp of 7 °C/min to 220 °C (held for 5 min), resulting in a total run time of 39 min. Mass spectrometric conditions were set as follows: electron impact (EI) ionization at 70 eV; ion source temperature of 250 °C; quadrupole temperature of 150 °C; transfer line temperature of 220 °C; and a mass scan range of m/z 35–500 with a scan rate of 3.6 scans/s. For sample preparation, 5 mL of green plum wine was transferred into a 20 mL headspace vial, followed by the addition of 1.5 g sodium chloride (Chengdu Kelong Chemical Co., Ltd., Chengdu, China) and 20 μL of internal standard solution (amyl acetate (Anpel Laboratory Technologies (Shanghai) Inc., Shanghai, China), 1 mL/L in anhydrous ethanol (Chengdu Kelong Chemical Co., Ltd., Chengdu, China) ). The vial was immediately sealed and equilibrated at 45 °C for 15 min. Volatile compounds were extracted using the preconditioned SPME fiber at 45 °C for 30 min, followed by thermal desorption in the GC injector at 210 °C for 5 min. All analyses were performed in triplicate to ensure reproducibility. Volatile compounds were identified by comparison of mass spectra with the NIST 17 (National Institute of Standards and Technology, Gaithersburg, MD, USA) mass spectral library, combined with retention index (RI) matching for further confirmation. Semiquantitative analysis was conducted using the internal standard method, and results were expressed as relative concentrations based on peak area ratios to the internal standard [22]. The validation parameters for GC–MS analysis are provided in the Supplementary Materials.

### 2.4. Odor Activity Value (OAV) Analysis

Odor activity value (OAV) is widely used to quantitatively evaluate the contribution of individual volatile compounds to the overall aroma profile. It is defined as the ratio of the concentration of a compound to its corresponding odor threshold. Compounds with OAV ≥ 1 are generally considered to contribute significantly to the overall aroma [23].

### 2.5. Construction of a Flavor Model for Green Plum Wine

**Figure 1:**
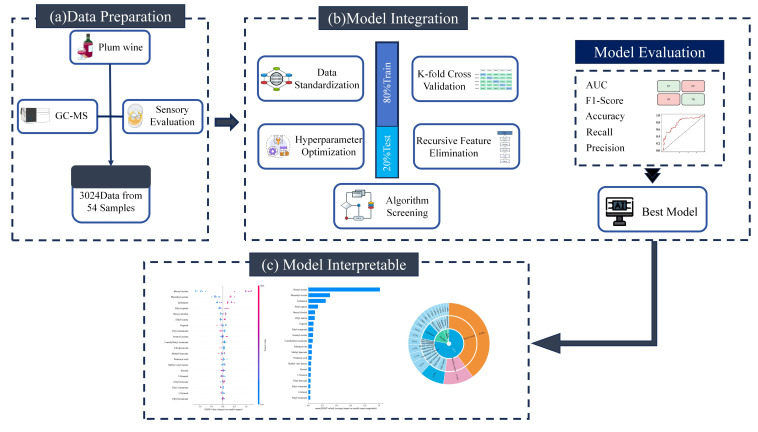
Machine Learning model flowchart.

To systematically evaluate the performance of different algorithms and identify the most suitable model for flavor classification, six clustering algorithms and six machine learning (ML) models were implemented and comparatively analyzed. The overall workflow of the ML analysis is presented in Figure 1. All models were implemented and evaluated using MATLAB R2022a.

#### 2.5.1. Construction of a Flavor Cluster Model for Green Plum Wine

Six clustering algorithms were employed to construct the flavor clustering model, including K-means clustering, hierarchical clustering analysis (HCA), Gaussian mixture model (GMM), fuzzy C-means (FCM), Canopy, and density-based spatial clustering of applications with noise (DBSCAN). Prior to clustering, the flavor component data were normalized using Z-score standardization to eliminate scale effects. Principal component analysis (PCA) was subsequently applied for dimensionality reduction to minimize noise and reduce the risk of overfitting, thereby improving model generalization [24]. The specific parameter settings were as follows: squared Euclidean distance was used as the clustering metric; for the Canopy algorithm, the threshold was set to 0.005 to ensure representative cluster centers; and for the DBSCAN algorithm, the neighborhood radius and minimum number of samples were set to 1 and 3, respectively.

To quantitatively evaluate the performance of different clustering algorithms, the Silhouette Coefficient (SC), Davies–Bouldin Index (DBI), and Calinski–Harabasz Index (CHI) were adopted as evaluation metrics. The SC measures clustering compactness and separation by comparing the average distance between each sample and its assigned cluster with that to other clusters, with higher values indicating better clustering performance. The DBI assesses the ratio of intra-cluster dispersion to inter-cluster separation, where lower values indicate superior clustering quality. The CHI evaluates clustering performance by calculating the ratio of between-cluster dispersion to within-cluster dispersion, with higher values reflecting stronger intra-cluster cohesion and greater inter-cluster separation [25].

#### 2.5.2. Construction of a Flavor Classification Model for Green Plum Wine

For flavor classification of green plum wine, six machine learning (ML) algorithms were employed, including k-nearest neighbor (KNN), naive Bayes (NB), random forest (RF), decision tree (DT), support vector machine (SVM), and a hybrid multilayer perceptron–random forest (MLP–RF) model [26]. The dataset was randomly divided into training and test sets at a ratio of 8:2 using a stratified sampling strategy. To improve model robustness, 10-fold cross-validation was performed during model training. Feature selection was conducted using recursive feature elimination with cross-validation (RFE-CV). The detailed hyperparameters are provided in the Supplementary Materials.

Model performance was comprehensively evaluated using five metrics: accuracy, precision, recall, F1 score, and the area under the receiver operating characteristic curve (AUC). All metrics range from 0 to 1, with higher values indicating superior model performance [27]. To enhance model interpretability, the SHapley Additive exPlanations (SHAP) framework was applied for global feature importance analysis. SHAP values were calculated to quantify the contribution of each feature to model predictions, thereby improving the transparency and reliability of the model [28].

### 2.6. Statistical Analysis

Statistical analyses were performed using SPSS 27.0 (IBM Corp., Armonk, NY, USA). Differences among sample groups were evaluated by one-way analysis of variance (ANOVA), followed by Tukey’s honestly significant difference (HSD) test for post hoc multiple comparisons. A *p*-value < 0.05 was considered statistically significant. All quantitative data are expressed as mean ± standard deviation ($\bar{x}$ ± SD) [29]. In addition, Origin 2024 (OriginLab, Northampton, MA, USA) and the Python 3.10 programming language were used for supplementary analyses and data visualization.

## 3. Results and Discussion

### 3.1. Sensory Quantitative Descriptive Analysis

**Figure 2:**
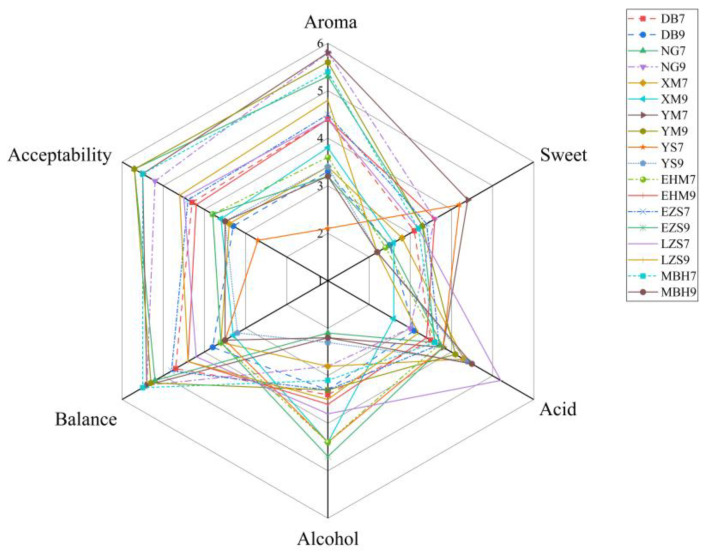
Sensory evaluation results of green plum wine samples by quantitative descriptive analysis.

A sensory evaluation of 18 green plum wine samples was conducted using quantitative descriptive analysis (QDA), and the results are presented in Figure 2. In terms of aroma characteristics, samples NG9, YM7, and YM9 exhibited the most pronounced floral and fruity notes, followed by MBHM7 and NG7. This distinctive sensory profile is likely associated with their relatively higher concentrations of ester-derived volatile compounds, including ethyl octanoate, ethyl hexanoate, methyl benzoate, and methyl 2-hydroxybenzoate. These esters impart pleasant fruity and floral notes. Consistently, these samples contained a relatively diverse range of ester compounds, which may contribute to increased aroma complexity [30]. Regarding taste attributes, sample LZS7 exhibited significantly higher acidity, which may be attributed to elevated levels of naturally occurring organic acids, such as citric and malic acids, in the raw fruit. Overall, the fermented green plum wines YM9, NG7, MBHM7, and YM7 demonstrated superior sensory performance across multiple attributes, particularly in terms of floral and fruity aroma intensity, flavor balance, and overall acceptability. Notably, wines produced from green plums sourced from different geographical regions exhibited distinct aroma profiles and sensory characteristics. Such variations highlight the significant influence of geographical origin and raw material composition on sensory attributes and quality differentiation of green plum wine [31]. The study protocol, including all sensory evaluation procedures, was formally approved by the Ethics Review Committee of Sichuan University of Science and Engineering (IRB Approval No. 2025LLSC022).

### 3.2. Analysis of Aroma Components and OAV in Different Varieties of Green Plum Wine

**Figure 3:**
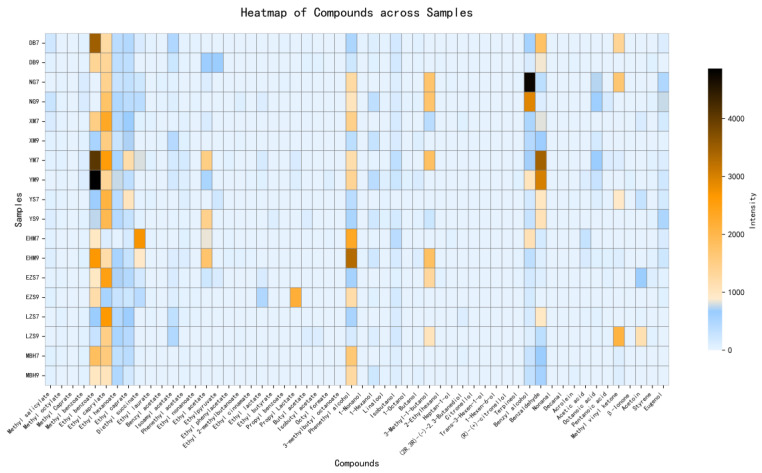
Heatmap of aroma compound concentrations in fermented green plum wine from different varieties.

Aroma is a key determinant of the sensory quality of fruit wines and is primarily governed by the presence and concentration of volatile compounds, such as esters, higher alcohols, and volatile fatty acids, which collectively shape the aroma profile of fermented wines [32]. In this study, HS-SPME–GC–MS was employed to characterize the volatile constituents of 18 green plum wine samples (Figure 3). A total of 56 aroma-active compounds were identified, including 27 esters, 17 alcohols, 3 acids, 4 aldehydes, 3 ketones, and 2 other volatile compounds, as summarized in Table 2. Substantial variations were observed in both the composition and concentration of these compounds among different varieties. Esters were the most abundant class in nearly all samples. For instance, ethyl benzoate, ethyl octanoate, and diethyl butanedioate reached maximum concentrations of 4820.53 μg/L, 2640.83 μg/L, and 2791.57 μg/L, respectively. These compounds typically impart pleasant floral, fruity, and sweet notes, thereby enhancing the overall aromatic intensity of green plum wine [3]. Among alcohols, 2-phenylethanol exhibited relatively high concentrations in several samples, reaching 1244.41 μg/L in NG7 and 1435.95 μg/L in XM7. This compound imparts a rose-like aroma and contributes to the roundness and complexity of the overall aroma profile. [33]. In addition, benzenecarbaldehyde was detected at elevated levels in certain samples, such as 3432.96 μg/L in YM7, likely imparting almond- and stone fruit-like notes. The coexistence of esters (e.g., ethyl hexanoate) and aldehydes (e.g., benzenecarbaldehyde) may result in synergistic aromatic effects, contributing to the complex floral, fruity, and sweet characteristics of fermented green plum wine [34]. In summary, these compounds were among the predominant volatile constituents in fermented green plum wine, potentially contributing to its characteristic aroma profile.

Additionally, OAV analysis identified 13 key odor-active compounds (OAV > 1), including seven esters, two alcohols, two ketones, and two compounds from other chemical classes, highlighting their significant contribution to the overall aroma profile. Among these, ethyl octanoate, methyl benzoate, ethyl hexanoate, methyl 2-hydroxybenzoate, 3-methylbutyl ethanoate, hexan-1-ol, and 3,7-dimethylocta-1,6-dien-3-ol exhibited the highest OAVs, suggesting that they play a decisive role in defining the fruity and floral characteristics of green plum wine. Meanwhile, methyl octanoate, ethyl decanoate, phenylmethanol, benzenecarbaldehyde, and octanoic acid exhibited OAVs between 0.1 and 1. Compounds within this range may exert synergistic effects and contribute to the overall aroma profile of fruit wines [35]. Although these compounds did not exceed the OAV threshold (≥1) for direct aroma contribution, they may still influence the overall aroma through synergistic or additive interactions, thereby affecting the perceived balance and harmony of green plum wine [36]. Overall, the aroma profile of green plum wine is predominantly driven by esters and alcohols with high OAVs, whereas compounds with moderate OAVs act as important secondary contributors, enhancing aroma depth and complexity. This finding highlights the diversity and synergistic interactions among volatile constituents in shaping the characteristic flavor profile of green plum wine.

### 3.3. Machine Learning-Based Flavor Clustering Model

**Figure 4:**
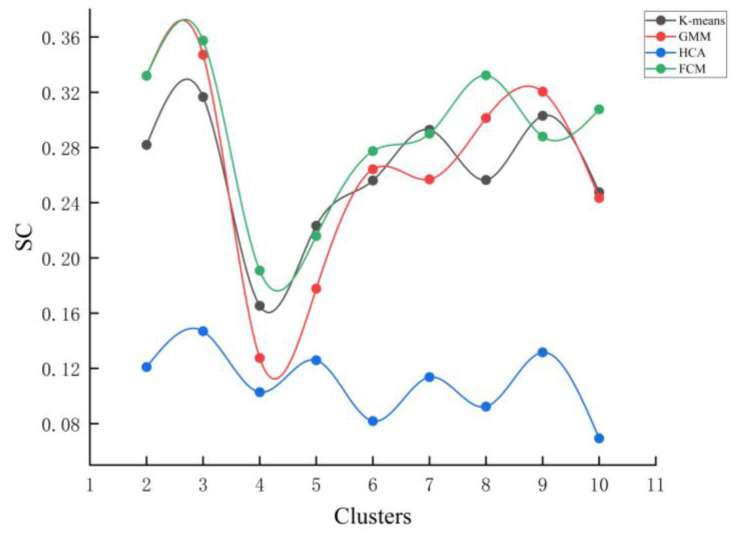
K-means, GMM, HCA, and FCM algorithm cluster contour coefficient diagram. Note: clusters = number of clusters. SC = silhouette coefficient (higher is better).

**Figure 5:**
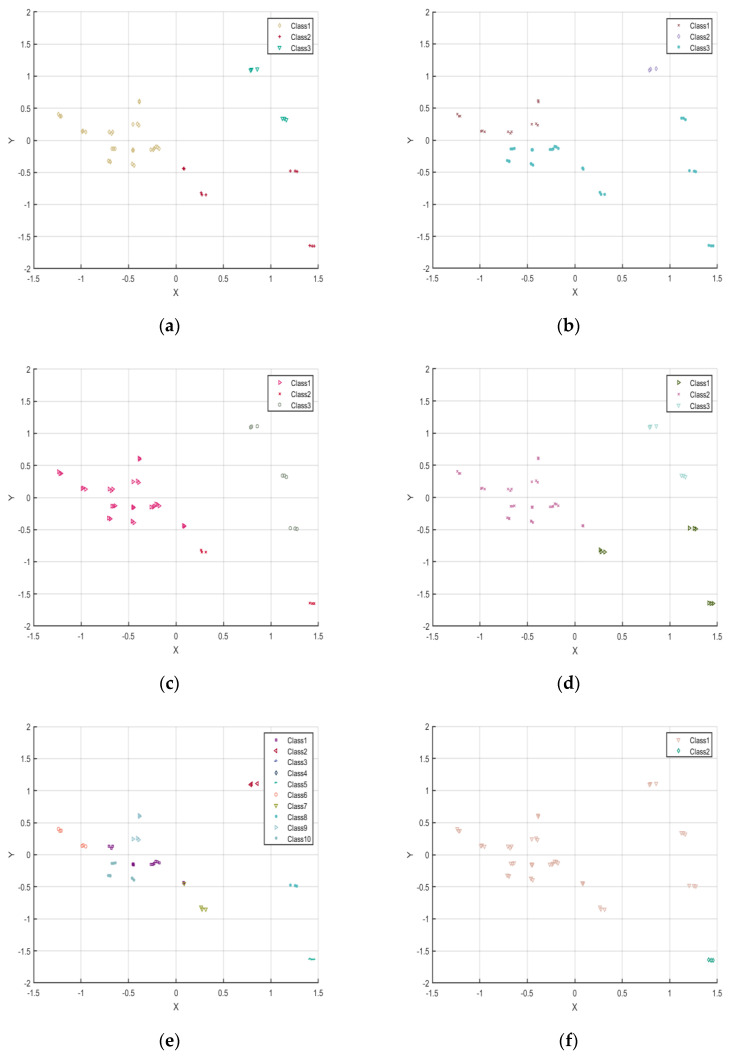
(**a**) K-means cluster data distribution map. (**b**) HCA cluster data distribution map. (**c**) GMM cluster data distribution map. (**d**) FCM cluster data distribution map. (**e**) Canopy cluster data distribution map. (**f**) DBSCAN cluster data distribution map. Note: Class indicates the number of clusters under different algorithms.

As shown in Figure 4, the optimal number of clusters for k-means, HCA, GMM, and FCM was determined to be K = 3. Among these algorithms (Table 3), FCM demonstrated the best overall performance, with SC = 0.35746, DBI = 1.753, and CHI = 8.7657, indicating a strong ability to discriminate between samples while maintaining high intra-cluster compactness and clear inter-cluster separation. In contrast, HCA exhibited relatively poor clustering performance at the same K value (SC = 0.14688, DBI = 2.2316, CHI = 6.6214), suggesting its limited suitability for the present flavor dataset. Following parameter optimization, Canopy and DBSCAN achieved relatively strong clustering performance. Specifically, Canopy yielded SC = 0.41999, DBI = 1.2279, and CHI = 10.3036, whereas DBSCAN produced SC = 0.39817, DBI = 0.68536, and CHI = 6.4063. Despite these favorable statistical metrics, both algorithms showed limited interpretability in terms of practical flavor classification. In particular, Canopy identified an optimal cluster number of 10, whereas DBSCAN produced only two clusters. Such excessively large or small cluster numbers may improve statistical indices but reduce the practical interpretability and applicability of classification results in a sensory context. As illustrated in Figure 5, the samples exhibited clear separation in the reduced-dimensional feature space, supporting the rationality of selecting three clusters. Considering both clustering performance and interpretability, FCM demonstrated the most balanced and robust performance for green plum wine flavor classification. In summary, the FCM algorithm with K = 3 was identified as the optimal model, providing a reliable and objective basis for classifying the flavor profiles of green plum wine.

**Table 3:** Performance Metrics for Six Clustering Algorithms.

Performance Metrics	K-means	HCA	GMM	FCM	Canopy	DBSCAN
SC	0.31664	0.14688	0.34714	0.35746	0.41999	0.39817
DBI	1.9357	2.2316	1.8589	1.753	1.2279	0.68536
CHI	8.5772	6.6214	8.0457	8.7657	10.3036	6.4063

Note: The higher the SC value, the better the clustering performance. The lower the DBI value, the better the clustering quality. The higher the CHI value, the better the separation effect.

### 3.4. Machine Learning-Based Flavor Classification Model

**Figure 6:**
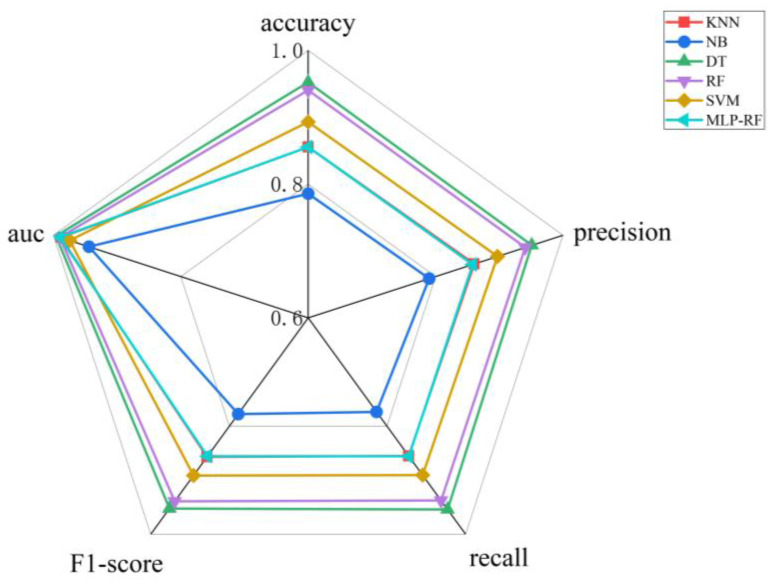
Radar Chart of Performance Metrics for Six Classification Algorithms.

**Figure 7:**
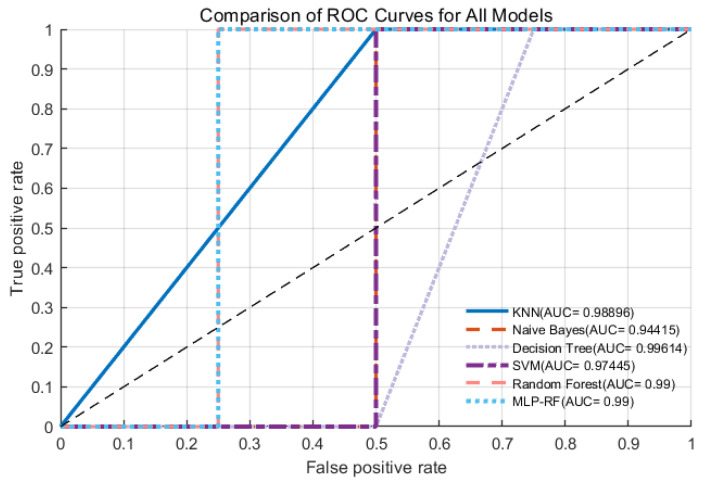
Macro ROC Curves for All Models.

**Figure 8:**
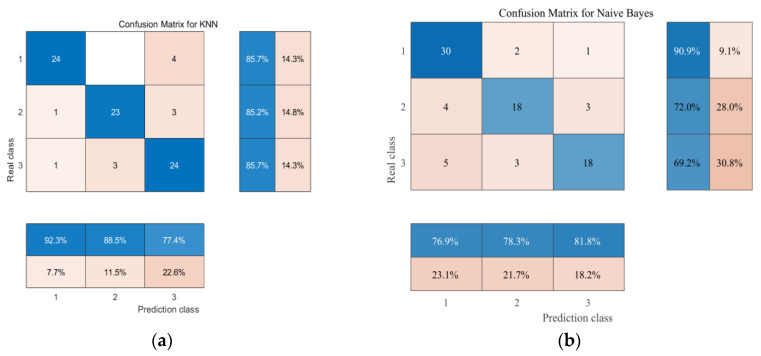

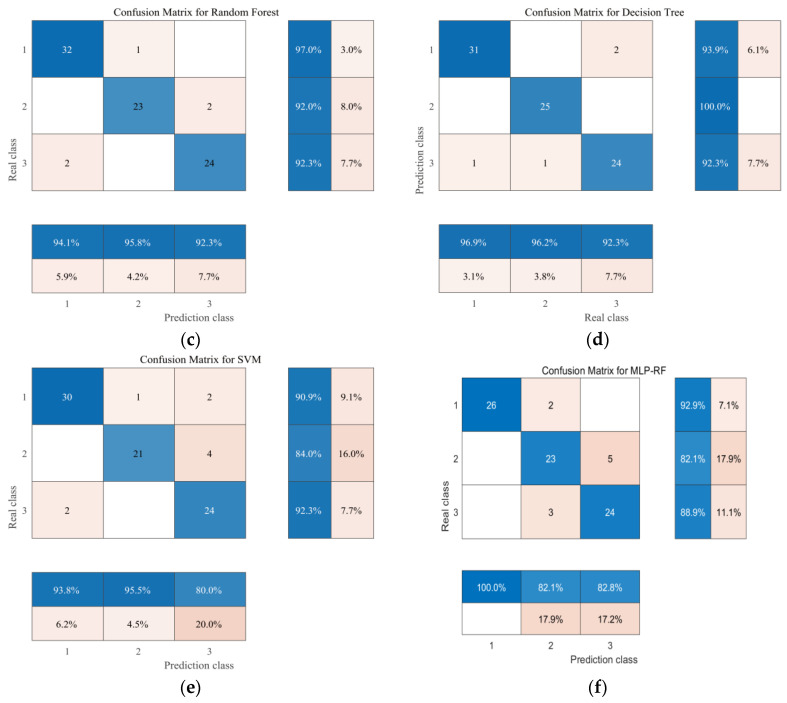
(**a**) Confusion Matrix for the KNN Model. (**b**) Confusion Matrix for the Naive Bayes Model. (**c**) Confusion Matrix for the Random Forest Model. (**d**) Confusion Matrix for the Decision Tree Model. (**e**) Confusion Matrix for the Support Vector Machine Model. (**f**) Confusion Matrix for the Multilayer Perceptron–Random Forest Model.

As illustrated in Figure 6, the radar chart comparing the six classification models shows that all algorithms achieved AUC values greater than 0.8, indicating good predictive performance for the flavor classification of green plum wine [37]. Specifically, the decision tree (DT) model outperformed the other algorithms across all key metrics, as reflected by the largest coverage area in the radar chart, indicating strong and well-balanced predictive capability. In contrast, the naive Bayes (NB) model exhibited the smallest coverage area, consistent with its comparatively lower performance. Among the six models, the DT model demonstrated the highest predictive performance (AUC > 0.99). Based on comparisons of confusion matrices, ROC curves, and overall performance metrics, the DT model was identified as the optimal algorithm for constructing the flavor prediction model of green plum wine. The best-performing DT model achieved an accuracy of 95.13% and an F1-score of 0.95244, whereas the NB model showed the lowest performance, with an accuracy of 78% and an F1-score of 0.77778. This difference may be attributed to correlations among input features, which can violate the independence assumption of the NB algorithm and thereby limit its classification performance. In terms of recall (Figure 7), all six algorithms achieved values above 0.7. The DT, random forest (RF), and support vector machine (SVM) models maintained recall values above 0.9 across all classes, indicating strong reliability in minimizing misclassification and accurately identifying each flavor category. Overall, all six models demonstrated satisfactory performance in predicting the flavor classification of green plum wine (Figure 8), likely due to their ability to capture complex relationships among variables [38]. Notably, the current dataset is limited to green plum wine samples produced under specific fermentation conditions, which may restrict the generalizability of the model to other fruit wines or processing conditions. In addition, although the DT model is highly interpretable, it may be prone to overfitting, particularly with small datasets, and its performance may decline when applied to samples with flavor profiles outside the training distribution.

### 3.5. Interpretable Machine Learning: SHAP Feature Analysis

**Figure 9:**
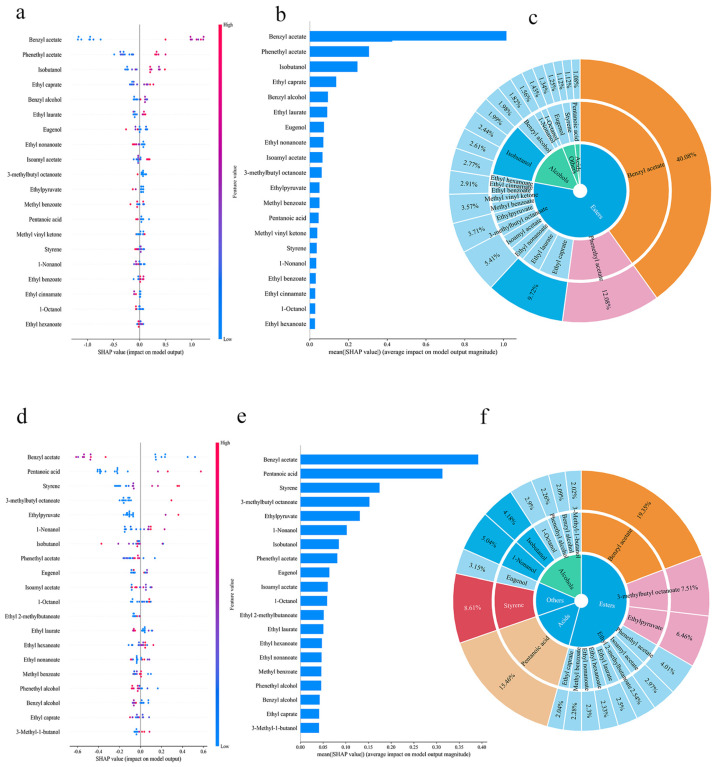

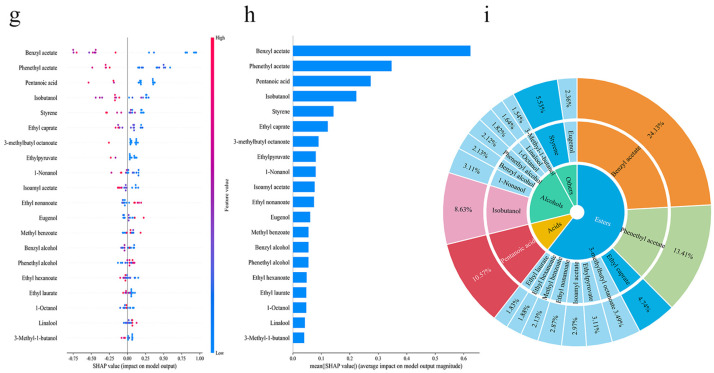
SHAP beeswarm plots ranking feature importance in sensory quality grade prediction models: (**a**) Class 1; (**d**) Class 2; (**g**) Class 3. SHAP bar chart illustrates the impact of different classifications on the sensory quality grading classification model: (**b**) Class 1; (**e**) Class 2; (**h**) Class 3. Sunrise Plot of SHAP Importance Variable Distribution: (**c**) Class 1; (**f**) Class 2; (**i**) Class 3.

To enhance the transparency and interpretability of the machine learning models, the SHapley Additive exPlanations (SHAP) method was applied to rank and interpret the importance of key volatile compounds influencing the flavor classification of green plum wine. This analysis was conducted based on the decision tree (DT) model, which exhibited the best predictive performance among the six algorithms. As illustrated in Figure 9, the model predictions for volatile compounds across different categories of green plum wine exhibited distinct patterns. The SHAP beeswarm plots (Figure 9a,d,g) indicate that higher concentrations of key compounds are associated with positive SHAP values, reflecting a positive contribution to model output, whereas lower concentrations are associated with negative contributions. In Category 1, volatile compounds such as benzyl ethanoate, 2-phenylethyl ethanoate, and 2-methylpropan-1-ol contributed substantially to the model output. In Category 2, benzyl ethanoate, pentanoic acid, and ethenylbenzene exhibited relatively higher contributions, with pentanoic acid and ethenylbenzene showing higher mean absolute SHAP values than other compounds. In Category 3, benzyl ethanoate, 2-phenylethyl ethanoate, pentanoic acid, and 2-methylpropan-1-ol also demonstrated notable contributions, with relatively balanced SHAP values among these variables. These differences suggest that the flavor characteristics of different categories are governed by distinct contribution patterns. Category 1 may be driven by a limited number of dominant compounds, whereas Categories 2 and 3 may reflect more distributed and synergistic contributions from multiple volatile components. In addition, benzyl ethanoate and 2-phenylethyl ethanoate were consistently identified as important features, which are associated with their characteristic aroma properties [39,40,41].

Meanwhile, the key flavor compounds identified through SHAP analysis showed strong consistency with those exhibiting high relative abundance in the preceding descriptive statistical analysis, as well as with key odor-active compounds with OAVs greater than 1. This consistency is further supported by the SHAP importance distribution sunburst plots (Figure 9c,f,i), where esters account for the largest proportion of volatile compounds (over 55% in all categories), consistent with their dominant SHAP contributions. In contrast, alcohols and acids exhibit category-specific variations in abundance that correspond to their respective SHAP importance. As illustrated in Figure 9, the SHAP value distribution of key volatile compounds highlights the predominance of esters and alcohols. This suggests that ester content plays a critical role in model-based flavor classification, with even minor variations in the concentration of certain esters potentially leading to perceptible changes in flavor attributes. For example, benzyl ethanoate shows a high contribution in Category 1, with high-concentration regions in the SHAP beeswarm plots corresponding to strong positive SHAP values. Moderate levels of benzyl ethanoate contribute to enhanced fruity and floral notes. Similarly, 2-methylpropan-1-ol exhibits a high contribution in Category 3, where elevated concentrations are associated with positive SHAP values, confirming its role as a key contributor to this category. This compound is associated with increased astringency and improved mouthfeel. Overall, the combined positive SHAP contributions of esters and alcohols reflect their synergistic effects on flavor perception, while variations in acid contributions across categories may be related to their modulatory role in overall flavor perception. In summary, SHAP-based interpretability analysis indicates that the flavor-driving compounds identified by the DT model effectively capture the characteristic sensory profile of green plum wine. These are predominantly fruity esters, complemented by floral and mellow notes, collectively forming a harmonious, multilayered, and balanced aroma profile.

## 4. Conclusions

This study integrates HS-SPME–GC–MS, OAV analysis, multivariate clustering, machine learning, and SHAP-based interpretability to systematically characterize the aroma profile and flavor classification of fermented green plum wine. The results demonstrate that the aroma profile is dominated by esters, accompanied by alcohols and minor acidic and aldehydic compounds, collectively contributing to a complex flavor matrix. Multivariate clustering analysis revealed that the flavor space of green plum wine can be structured into three distinct categories. Among the evaluated models, the decision tree achieved the best balance between predictive accuracy and interpretability, while fuzzy c-means clustering provided the most consistent and meaningful partitioning of flavor categories. SHAP-based interpretability analysis, combined with OAV evaluation, identified benzyl ethanoate and 2-phenylethyl ethanoate as key contributors to the sensory attributes of green plum wine, further demonstrating the consistency between chemical composition and sensory perception. This study provides new insights into the relationship between volatile composition and sensory perception, demonstrating the utility of interpretable machine learning for elucidating key drivers of complex flavor systems. The proposed framework offers a rapid and objective strategy for flavor evaluation and classification, with potential applications in quality control, product standardization, and process optimization in fruit wine production. However, this study is limited by the relatively small dataset and restricted fermentation conditions, which may affect the generalizability of the model. Future work should expand sample diversity across different fruit varieties and fermentation environments and further improve model robustness to enhance its applicability in broader production scenarios.

## Figures and Tables

**Table 2:** Identification of volatile compounds in 18 types of green plum wine using HS-SPME–GC–MS.

Compound Name	ThreshOld (μg/L)	Mass Concentration (μg/L)																	
		DB7	DB9	NG7	NG9	XM7	XM9	YM7	YM9	YS7	YS9	EHM7	EHM9	EZS7	EZS9	LZS7	LZS9	MBHM7	MBHM9
Methyl 2-hydroxybenzoate	34.00	195.26 ± 3.97 ^b^	46.38 ± 0.79 ^h^	-	249.53 ± 8.09 ^a^	115.46 ± 3.38 ^c^	43.04 ± 0.31 ^h^	46.5 ± 0.52 ^h^	-	61.64 ± 0.81 ^f^	95.37 ± 1.19 ^d^	46.97 ± 2.75 ^h^	87.57 ± 1.23 ^e^	25.95 ± 1.47 ^i^	61.17 ± 5.24 ^f^	14.57 ± 1.59 ^j^	11.82 ± 1.93 ^j^	62.85 ± 2.30 ^f^	55.49 ± 0.84 ^g^
Methyl octylate	200.00	12.75 ± 0.38 ^j^	13.77 ± 1.25 ^j^	28.62 ± 0.67 ^d^	25.29 ± 0.54 ^ef^	23.81 ± 1.05 ^fg^	20.3 ± 0.46 ^h^	50.31 ± 1.61 ^a^	22.82 ± 0.45 ^g^	26.53 ± 1.65 ^de^	22 ± 1.04 ^gh^	-	-	31.8 ± 1.47 ^c^	14.23 ± 1.46 ^j^	38.69 ± 1.86 ^b^	26.78 ± 3.44 ^de^	22.2 ± 0.85 ^gh^	17.19 ± 0.80 ^i^
Methyl decanoate	-	6.87 ± 0.39 ^d^	4.02 ± 0.26 ^g^	-	-	10.57 ± 0.94 ^bc^	9.87 ± 0.60 ^c^	-	-	12.29 ± 1.02 ^a^	-	-	-	5.67 ± 0.28 ^ef^	-	11.18 ± 0.54 ^b^	5.03 ± 1.73 ^f^	6.38 ± 0.48 ^de^	-
Methyl benzoate	0.50	49.21 ± 1.59 ^f^	30.2 ± 1.01 ^j^	133.91 ± 1.46 ^a^	128.93 ± 2.51 ^b^	29.37 ± 2.08 ^j^	38.79 ± 0.54 ^i^	109.47 ± 3.91 ^c^	112.82 ± 2.67 ^c^	18.72 ± 0.44 ^k^	40.59 ± 0.10 ^hi^	28.41 ± 2.33 ^j^	67.78 ± 5.90 ^d^	21.79 ± 0.25 ^k^	14.5 ± 0.61 ^l^	42.4 ± 2.05 ^ghi^	46.04 ± 1.16 ^fg^	62.09 ± 1.21 ^e^	42.93 ± 0.38 ^gh^
Ethyl benzoate	1433.65	3506.31 ± 26.61 ^c^	1342.88 ± 14.41 ^g^	-	-	1503.99 ± 35.78 ^f^	541.79 ± 13.24 ^l^	4091.52 ± 21.55 ^b^	4820.53 ± 89.24 ^a^	626.61 ± 2.08 ^k^	744.05 ± 2.61 ^j^	955.28 ± 27.73 ^i^	2659.78 ± 9.91 ^d^	980.13 ± 21.21 ^i^	1193.9 ± 10.65 ^h^	641.26 ± 22.92 ^k^	279.76 ± 2.11 ^m^	1801.37 ± 25.25 ^e^	974.38 ± 6.96 ^i^
Ethyl octanoate	12.87	1244.26 ± 36.29 ^k^	1349.28 ± 28.92 ^j^	1458.42 ± 36.11 ^i^	1748.22 ± 13.48 ^f^	2278.36 ± 115.28 ^c^	1545.94 ± 25.62 ^gh^	2633.79 ± 28.18 ^a^	1366.59 ± 21.04 ^j^	2077.14 ± 19.46 ^d^	1972.26 ± 3.7 ^e^	99.85 ± 5.12 ^n^	1202.48 ± 9.5 ^k^	2532.17 ± 42.55 ^b^	564.17 ± 18.71 ^m^	2640.83 ± 24.16 ^a^	1487.15 ± 3.84 ^hi^	1567.92 ± 31.16 ^g^	1046.31 ± 10.93 ^l^
Ethyl hexanoate	14.00	392.68 ± 8.86 ^g^	362.55 ± 5.73 ^h^	273.65 ± 1.10 ^j^	461.83 ± 0.76 ^e^	423.56 ± 27.94 ^f^	252.34 ± 1.17 ^k^	515.36 ± 3.04 ^d^	772.02 ± 7.70 ^a^	376.42 ± 4.86 ^gh^	430.73 ± 1.63 ^f^	27.99 ± 3.42 ^l^	545.27 ± 2.21 ^c^	714.73 ± 5.62 ^b^	284.61 ± 9.95 ^j^	420.32 ± 20.73 ^f^	543.58 ± 25.11 ^c^	335.52 ± 3.76 ^i^	464.06 ± 2.60 ^e^
Ethyl decanoate	1122.30	422.41 ± 21.47 ^f^	358.37 ± 6.67 ^h^	316.68 ± 2.27 ^i^	381.39 ± 1.13 ^g^	656.27 ± 26.86 ^d^	711.04 ± 4.44 ^c^	1179.48 ± 25.87 ^a^	353.53 ± 4.98 ^h^	1039.94 ± 20.63 ^b^	294.42 ± 1.21 ^j^	134.08 ± 3.00 ^m^	285.43 ± 2.53 ^j^	457.42 ± 3.08 ^e^	230.37 ± 4.39 ^k^	656.76 ± 23.14 ^d^	431.84 ± 2.34 ^f^	392.14 ± 4.22 ^g^	180.72 ± 4.40 ^l^
Diethyl butanedioate	353,193.25	126.79 ± 5.07 ^f^	-	213.15 ± 1.84 ^e^	402.16 ± 12.50 ^d^	57.78 ± 2.61 ^g^	-	798.75 ± 7.85 ^c^	-	32.08 ± 0.90 ^gh^	50.73 ± 1.21 ^g^	2791.57 ± 91.64 ^a^	918.61 ± 7.29 ^b^	177.89 ± 4.00 ^e^	431.82 ± 19.15 ^d^	8.39 ± 1.12 ^h^	3.44 ± 0.10 ^h^	34.9 ± 1.30 ^gh^	21.22 ± 1.84 ^gh^
Ethyl dodecanoate	1,200,000.00	26.85 ± 2.06 ^d^	13.3 ± 0.72 ^f^	-	15.55 ± 0.12 ^f^	35.7 ± 2.84 ^c^	-	70.2 ± 1.55 ^a^	-	54.33 ± 0.74 ^b^	-	21.39 ± 0.46 ^e^	-	-	24.63 ± 4.16 ^d^	20.48 ± 2.00 ^e^	15.45 ± 0.55 ^f^	-	-
Benzyl ethanoate	-	13.61 ± 0.96 ^f^	3.28 ± 0.23 ^j^	40.22 ± 0.45 ^a^	25.97 ± 0.73 ^b^	9.07 ± 0.52 ^h^	20.82 ± 0.59 ^c^	17.27 ± 0.68 ^d^	14.28 ± 1.29 ^f^	5.5 ± 0.60 ^i^	-	15.5 ± 1.79 ^e^	-	-	2.97 ± 0.28 ^j^	-	11.24 ± 0.94 ^g^	-	-
3-Methylbutyl ethanoate	93.93	452.75 ± 28.61 ^b^	237.84 ± 0.52 ^e^	48.31 ± 0.47 ^i^	76.47 ± 2.68 ^h^	20.62 ± 0.76 ^j^	423.2 ± 3.85 ^c^	166.58 ± 4.80 ^f^	172.16 ± 1.66 ^f^	-	35.18 ± 0.66 ^ij^	-	111.08 ± 0.95 ^g^	112.55 ± 5.60 ^g^	98.27 ± 3.28 ^g^	342.87 ± 17.70 ^d^	492.85 ± 20.64 ^a^	-	-
2-Phenylethyl ethanoate	909.00	31.4 ± 1.00 ^g^	22.35 ± 1.82 ^hi^	-	-	47.94 ± 3.37 ^e^	67.55 ± 3.08 ^d^	151.41 ± 4.63 ^a^	76.3 ± 2.61 ^c^	74.92 ± 1.42 ^c^	34.55 ± 1.28 ^g^	73.94 ± 0.37 ^c^	97.43 ± 3.03 ^b^	25.53 ± 2.31 ^h^	21.78 ± 2.02 ^hi^	39.67 ± 2.55 ^f^	18.59 ± 1.31 ^i^	34.06 ± 0.89 ^g^	24.37 ± 2.77 ^h^
Ethyl nonanoate	3150.61	24.6 ± 0.85 ^c^	8.95 ± 1.63 ^g^	-	38.34 ± 1.50 ^a^	-	-	-	-	-	-	-	-	10.54 ± 0.31 ^f^	26.25 ± 2.23 ^b^	21 ± 1.50 ^d^	5.11 ± 0.22 ^h^	-	17.6 ± 1.42 ^e^
Ethyl ethanoate	32,551.60	-	641.02 ± 6.80 ^e^	113.61 ± 1.95 ^h^	-	117.39 ± 6.69 ^h^	-	1536.95 ± 29.05 ^b^	515.43 ± 8.11 ^f^	-	1441.5 ± 14.13 ^c^	843.48 ± 7.78 ^d^	1684.16 ± 27.22 ^a^	217.16 ± 3.84 ^g^	-	-	-	-	-
Ethyl 2-oxopropanoate	-	-	654.19 ± 3.30 ^a^	-	-	-	-	-	-	192.22 ± 1.80 ^b^	-	-	-	125.2 ± 2.55 ^c^	-	-	-	-	-
Ethyl 2-phenylethanoate	406.83	-	-	-	-	-	-	47.42 ± 5.84 ^a^	-	-	-	35.12 ± 0.52 ^c^	41.32 ± 1.62 ^b^	-	-	-	-	-	-
Ethyl 2-methylbutanoate	18.00	-	-	-	81.74 ± 0.76 ^a^	-	28.62 ± 0.45 ^c^	-	-	-	-	-	43.31 ± 0.97 ^b^	14.43 ± 0.51 ^e^	-	-	19.13 ± 0.84 ^d^	-	-
Ethyl cinnamate	48.00	-	-	64.39 ± 83.86 ^a^	-	-	-	-	31.64 ± 2.72 ^b^	-	-	-	22 ± 1.73 ^b^	-	-	-	-	-	-
Ethyl 2-hydroxypropanoate	128,083.80	-	-	-	112.28 ± 5.30 ^c^	-	-	152.5 ± 1.90 ^b^	70.7 ± 0.41 ^d^	-	-	-	-	123.83 ± 4.86 ^c^	443.63 ± 31.98 ^a^	-	-	-	-
Ethyl butanoate	81.50	-	-	-	-	-	-	-	-	-	82.76 ± 1.56 ^a^	-	-	-	-	-	15.94 ± 0.98 ^b^	-	-
Propyl benzoate	-	30.09 ± 1.62 ^a^	4 ± 0.24 ^c^	29.31 ± 0.94 ^b^	-	-	-	-	-	-	-	-	-	-	-	-	-	-	-
Propyl 2-hydroxypropanoate	-	-	-	-	-	-	-	128.63 ± 1.45 ^b^	87.51 ± 4.02 ^c^	-	-	-	-	-	2206.5 ± 67.55 ^a^	-	-	-	-
Butyl ethanoate	-	20.75 ± 0.80 ^d^	41.98 ± 1.44 ^c^	-	-	-	-	-	-	-	101.87 ± 1.85 ^a^	-	-	-	-	-	100.33 ± 2.15 ^b^	-	-
Isobutyl ethanoate	25.00	-	55.35 ± 3.29 ^b^	-	-	-	-	-	-	-	-	-	-	-	-	-	102.82 ± 5.26 ^a^	-	-
Octyl methanoate	-	7.72 ± 0.43 ^e^	-	-	-	-	-	-	73.56 ± 3.92 ^a^	-	17.01 ± 0.77 ^c^	23.88 ± 1.18 ^b^	-	-	-	-	8.65 ± 0.47 ^e^	-	10.68 ± 0.52 ^d^
3-methylbutyl octanoate	125.00	-	-	-	13.29 ± 0.95 ^a^	-	-	-	-	8.01 ± 0.65 ^b^	-	-	-	6.5 ± 0.12 ^c^	-	-	-	-	-
2-phenylethanol	28,922.73	498.29 ± 8.59 ^k^	220.31 ± 4.54 ^m^	1244.41 ± 1.53 ^f^	979.09 ± 56.56 ^i^	1435.95 ± 48.92 ^d^	365.96 ± 4.19 ^l^	1121.32 ± 32.31 ^h^	1388.26 ± 12.90 ^e^	385.83 ± 3.51 ^l^	527.87 ± 1.52 ^jk^	2315.83 ± 32.73 ^b^	3406.58 ± 71.33 ^a^	569.57 ± 10.83 ^j^	1187.8 ± 10.98 ^g^	519.26 ± 19.31 ^k^	146.01 ± 9.49 ^n^	1641.47 ± 12.04 ^c^	1282.27 ± 9.92 ^f^
1-Nonanol	806.43	24.32 ± 0.42 ^b^	16.61 ± 1.12 ^c^	7.23 ± 0.37 ^e^	6.8 ± 0.71 ^e^	4.7 ± 0.39 ^f^	3.89 ± 0.76 ^f^	-	12.39 ± 0.84 ^d^	2.05 ± 0.07 ^g^	5.01 ± 0.93 ^f^	12.55 ± 1.40 ^d^	30.51 ± 1.17 ^a^	-	-	4.11 ± 0.29 ^f^	1.91 ± 0.28 ^g^	-	2.67 ± 0.32 ^g^
1-Hexanol	5.60	78.84 ± 0.62 ^h^	75.78 ± 2.20 ^h^	12.98 ± 0.13 ^l^	355.31 ± 4.71 ^b^	-	293.84 ± 1.45 ^c^	28.5 ± 0.78 ^j^	379.2 ± 2.33 ^a^	15.05 ± 0.71 ^kl^	205.77 ± 0.83 ^e^	18.02 ± 1.61 ^k^	193.35 ± 2.28 ^f^	9.24 ± 1.02 ^m^	67.11 ± 3.64 ^i^	-	109.17 ± 0.73 ^g^	17.8 ± 0.65 ^k^	209.18 ± 2.11 ^d^
3,7-dimethylocta-1,6-dien-3-ol	6.00	35.73 ± 2.40 ^c^	16.38 ± 1.34 ^h^	-	22.07 ± 0.54 ^f^	14.1 ± 0.57 ^i^	24.48 ± 1.18 ^e^	-	86.59 ± 2.06 ^a^	30.18 ± 1.73 ^d^	66.54 ± 1.33 ^b^	-	-	4.41 ± 0.31 ^j^	-	16.23 ± 1.51 ^h^	19.19 ± 1.27 ^g^	19.59 ± 1.12 ^g^	30.14 ± 0.74 ^d^
2-methylpropan-1-ol	28,300.00	178.87 ± 9.27 ^d^	178.39 ± 0.41 ^d^	95.71 ± 1.18 ^h^	120.15 ± 3.11 ^g^	146.76 ± 3.71 ^f^	121.99 ± 0.59 ^g^	355.87 ± 4.52 ^b^	242.4 ± 3.13 ^c^	-	164.76 ± 2.16 ^e^	377.99 ± 15.37 ^a^	147.34 ± 3.84 ^f^	71.66 ± 1.77 ^i^	158.04 ± 3.74 ^e^	128.75 ± 5.03 ^g^	177.42 ± 6.70 ^d^	90.06 ± 2.41 ^h^	77.62 ± 2.17 ^i^
1-Octanol	900.00	6.84 ± 0.40 ^g^	5.05 ± 0.74 ^h^	7.23 ± 0.04 ^g^	19.17 ± 0.97 ^b^	-	14.75 ± 0.83 ^c^	-	64.52 ± 0.27 ^a^	4.86 ± 0.12 ^h^	-	-	13.47 ± 1.30 ^d^	-	-	-	9.37 ± 0.39 ^f^	-	11.01 ± 1.24 ^e^
Butan-1-ol	2733.35	38.89 ± 1.08 ^g^	34.59 ± 1.65 ^h^	-	53.61 ± 2.64 ^d^	-	91.95 ± 2.63 ^c^	-	119.55 ± 2.13 ^a^	-	51.23 ± 0.97 ^e^	-	97.58 ± 1.72 ^b^	-	30.49 ± 0.39 ^i^	-	30.7 ± 1.07 ^i^	-	48.08 ± 0.89 ^f^
3-Methylbutan-1-ol	30,000.00	-	-	1735.06 ± 18.31 ^c^	1744.67 ± 24.35 ^c^	379.9 ± 8.28 ^f^	-	1862.05 ± 33.36 ^a^	-	-	247.6 ± 3.26 ^g^	-	1821.35 ± 19.54 ^b^	1325.42 ± 22.08 ^d^	-	-	1040.35 ± 30.86 ^e^	-	-
2-ethylhexan-1-ol	198.00	-	19.62 ± 0.93 ^b^	10.12 ± 0.13 ^d^	-	-	12.04 ± 0.64 ^c^	-	-	-	-	-	-	46.76 ± 1.59 ^a^	-	-	-	-	-
Heptan-1-ol	1433.94	-	6.48 ± 1.16 ^c^	20.25 ± 0.35 ^b^	-	-	3.65 ± 0.19 ^d^	-	-	-	-	41.56 ± 1.95 ^a^	-	-	-	-	-	-	-
(2R,3R)-butane-2,3-diol	-	-	8.19 ± 1.34 ^d^	-	-	59.49 ± 2.99 ^b^	-	-	-	-	-	-	-	-	-	96.94 ± 0.43 ^a^	8.36 ± 1.92 ^d^	47.87 ± 1.49 ^c^	-
3,7-dimethyloct-6-en-1-ol	10.00	-	1.02 ± 0.06 ^b^	-	-	-	-	-	-	2.81 ± 0.09 ^a^	-	-	-	-	-	-	-	-	-
(E)-hex-3-en-1-ol	-	-	-	-	-	-	4.36 ± 0.56 ^b^	-	12.32 ± 0.39 ^a^	-	-	-	-	-	-	-	3.28 ± 0.27 ^c^	-	-
Hex-5-en-1-ol	-	-	-	-	15.73 ± 1.01 ^a^	-	7.4 ± 0.51 ^b^	-	-	-	-	-	-	-	-	-	-	-	-
(R)-3,7-dimethyloct-6-en-1-ol	-	-	-	-	-	-	5.53 ± 1.04 ^a^	-	-	2.7 ± 0.08 ^b^	-	-	-	-	-	-	-	-	-
2-(4-methylcyclohex-3-en-1-yl)propan-2-ol	300.00	-	2.7 ± 0.33 ^j^	22.13 ± 0.18 ^e^	-	-	6.92 ± 1.62 ^h^	26.02 ± 1.18 ^c^	58.75 ± 0.51 ^a^	4.22 ± 0.13 ^i^	-	16.07 ± 1.25 ^f^	31.29 ± 0.43 ^b^	24.13 ± 1.28 ^d^	8.84 ± 0.38 ^g^	-	-	3.67 ± 0.28 ^ij^	-
Phenylmethanol	3000.00	564.55 ± 38.40 ^e^	132.13 ± 6.37 ^kl^	4779.01 ± 19.36 ^a^	2899.48 ± 92.40 ^b^	484.5 ± 20.36 ^f^	429.13 ± 2.12 ^g^	702.9 ± 6.20 ^d^	1011.7 ± 15.77 ^c^	188.44 ± 0.92 ^j^	276.03 ± 3.77 ^i^	1054.62 ± 40.86 ^c^	355.15 ± 3.34 ^h^	173.31 ± 1.54 ^jk^	166.18 ± 5.40 ^jk^	136.94 ± 7.23 ^kl^	94.87 ± 6.37 ^l^	279.27 ± 1.82 ^i^	306.33 ± 2.93 ^i^
Benzenecarbaldehyde	4203.10	1769.76 ± 14.69 ^c^	862.67 ± 16.36 ^f^	378.28 ± 1.79 ^j^	93.99 ± 1.87 ^l^	811.65 ± 35.58 ^g^	656.43 ± 17.19 ^h^	3432.96 ± 23.32 ^a^	3033.36 ± 18.02 ^b^	978.33 ± 2.39 ^e^	1065.76 ± 4.81 ^d^	152.96 ± 3.06 ^k^	96.87 ± 3.49 ^l^	76.61 ± 2.80 ^l^	7.25 ± 0.52 ^m^	973.66 ± 17.55 ^e^	368.55 ± 3.07 ^j^	678.8 ± 3.33 ^h^	558.02 ± 3.28 ^i^
Nonanal	122.45	-	2.54 ± 0.08 ^c^	-	-	-	-	-	-	-	-	-	-	10.97 ± 0.17 ^a^	-	-	-	-	7.49 ± 0.74 ^b^
Decanal	3.00	-	1.67 ± 0.22 ^b^	-	-	-	-	-	-	3.29 ± 0.07 ^a^	-	-	-	-	-	-	-	-	-
Propenal	0.05	-	35.29 ± 0.92 ^b^	-	-	-	-	-	-	33.54 ± 0.83 ^c^	-	-	-	-	-	-	-	10.49 ± 0.85 ^d^	45.44 ± 0.72 ^a^
Ethanoic acid	160,000.00	-	-	-	-	-	-	-	111.55 ± 2.07 ^c^	-	-	283.15 ± 6.66 ^a^	121.53 ± 1.18 ^b^	-	9.59 ± 1.77 ^e^	36.6 ± 1.98 ^d^	-	-	-
Octanoic acid	2701.23	-	6.57 ± 0.43 ^j^	742.41 ± 4.90 ^a^	606.61 ± 7.07 ^c^	-	157.3 ± 5.55 ^e^	643.94 ± 2.55 ^b^	293.56 ± 8.62 ^d^	79.39 ± 1.25 ^f^	-	-	64.96 ± 1.27 ^g^	-	-	44.69 ± 0.53 ^h^	21.04 ± 1.15 ^i^	-	-
Pentanoic acid	-	-	-	-	153.96 ± 14.24 ^a^	-	-	93.94 ± 2.19 ^b^	-	25.14 ± 0.41 ^f^	-	-	72.82 ± 1.93 ^c^	50.82 ± 1.29 ^d^	-	-	32.38 ± 1.47 ^e^	-	-
But-3-en-2-one	-	1365.95 ± 35.96 ^c^	-	1690.42 ± 8.81 ^b^	-	-	-	109.98 ± 2.44 ^e^	-	927.83 ± 2.90 ^d^	-	-	94.16 ± 3.52 ^f^	-	-	-	2099.66 ± 8.37 ^a^	83.27 ± 2.47 ^f^	82.83 ± 1.81 ^f^
(3E)-4-(2,6,6-trimethylcyclohex-1-en-1-yl)but-3-en-2-one	0.01	13.9 ± 0.36 ^b^	14.48 ± 1.18 ^b^	-	-	-	-	-	70.06 ± 4.08 ^a^	-	-	-	-	-	-	-	15.32 ± 1.60 ^b^	-	15.41 ± 1.59 ^b^
3-hydroxybutan-2-one	259.00	-	-	-	-	127.51 ± 4.91 ^d^	-	-	-	297.96 ± 1.88 ^c^	-	-	-	674.11 ± 24.71 ^b^	7.64 ± 0.34	25.23 ± 3.06	1110.82 ± 78.16 ^a^	-	-
Phenylethene	6.50	-	72.61 ± 2.38 ^b^	-	43.41 ± 0.83 ^de^	-	16.06 ± 0.77 ^h^	49.35 ± 1.39 ^c^	34.07 ± 0.11 ^g^	-	40.48 ± 0.38 ^f^	-	41.69 ± 2.26 ^ef^	80.43 ± 2.35 ^a^	-	-	45.43 ± 4.93 ^d^	-	-
4-allyl-2-methoxyphenol	7.00	98.21 ± 0.78 ^f^	13.97 ± 0.93 ^j^	486.44 ± 1.42 ^c^	737.98 ± 55.52 ^a^	152.69 ± 7.21 ^e^	37.22 ± 1.79 ^hi^	98.3 ± 1.56 ^f^	197.93 ± 4.15 ^d^	152.22 ± 1.14 ^e^	514.24 ± 2.09 ^b^	118.43 ± 3.07 ^f^	212.71 ± 1.17 ^d^	39.79 ± 1.70 ^h^	68.59 ± 3.33 ^g^	11.86 ± 0.36 ^j^	10.08 ± 2.62 ^j^	15.2 ± 0.78 ^ij^	12.04 ± 1.60 ^j^

Note: Different lowercase letters indicate significant differences among the eighteen cultivars in the same row (*p* < 0.05). “-” indicates not detected.

## Data Availability

The original contributions presented in the study are included in the article/Supplementary Materials. Further inquiries can be directed to the corresponding authors.

## Supplementary Materials

The following supporting information can be downloaded at: https://www.mdpi.com/article/10.3390/foods15081342/s1, Table S1: Linear regression and validation parameters for GC-MS analysis; Table S2: Hyperparameters of machine learning models used in this study.
