# Supplementary material for: Machine Learning and SHAP Feature Analysis: Classification Model for Aroma Components in Green Plum Wine
Source: Foods. 2026 Apr 13;15(8):1342. doi: 10.3390/foods15081342 (PMC13115067; doi:10.3390/foods15081342)
Supplement: Supplementary file 1 [file foods-15-01342-s001.zip › foods-4214357-supplementary.pdf]

Caption:

Table S1 Linear regression and validation parameters for GC-MS analysis

Table S2 Hyperparameters of machine learning models used in this study

Table S1 Linear regression and validation parameters for GC-MS analysis

Analyte	Regression Equation	R^2	Linear Range (μg/L)	LOD (μg/L)	LOQ (μg/L)	RSD (n=6)
Pentyl acetate	y = 2139.857x - 21267.28	0.9996	24.35-980.63	1.51	5	4.71%

Table S2 Hyperparameters of machine learning models used in this study

Model Name	Model Abbreviation	Optimized Hyperparameters
K-Nearest Neighbor	KNN	n_neighbors = 3, weights = 'uniform', metric = 'euclidean'
Naive Bayes	NB	GaussianNB, var_smoothing = 1e-9
Random Forest	RF	n_estimators = 20, max_depth = 3, min_samples_split = 2, random_state = 42
Decision Tree	DT	criterion = 'gini', max_depth = 2, min_samples_split = 2, random_state = 42
Support Vector Machine	SVM	kernel = 'linear', C = 0.1, gamma = 'scale', random_state = 42
Multilayer Perceptron–Random Forest	MLP-RF	hidden_layer_sizes = (3,), activation = 'relu', solver = 'lbfgs', max_iter = 1000, random_state = 42

Instructions 1: Dataset Splitting and Validation Strategy

The dataset consisted of 18 samples and was divided into training and test sets at a ratio of 8:2 using a stratified sampling strategy to preserve the class distribution.To ensure reproducibility, the data were randomly shuffled prior to splitting, and a fixed random seed (random_state = 42) was applied.Due to the relatively small sample size, model training and hyperparameter optimization were performed using 10-fold cross-validation within the training set. This approach allows each sample to be used for both training and

internal validation within the cross-validation framework, thereby improving the robustness and reliability of model evaluation.Model performance was primarily assessed based on cross-validation results, while the independent test set was used for final performance evaluation.

Instructions 2：Criterion for Optimal Hyperparameter Selection

The hyperparameters of all machine learning models were optimized by taking the 10-fold cross-validation accuracy of the training set as the primary evaluation criterion, with the secondary criteria of minimizing the classification error and ensuring the model generalization ability. The grid search method was adopted to traverse the preset hyperparameter space, and the parameter combination with the best comprehensive performance was selected as the optimized hyperparameter (Table S2).

Instructions 3：Data Standardization Before Classification

Beyond the clustering step, z-score standardization was uniformly applied to all feature data before the classification modeling process.

Instructions 4：Overfitting Control Strategies

1.Feature dimensionality reduction： By analyzing the variance of feature data and the correlation between features and

classification labels, redundant and irrelevant features are removed, avoiding model fitting noise caused by excessive features.

2.Model structure simplification： For tree-based models (RF, DT) and neural network models (MLP-RF), the model complexity was controlled by limiting key parameters (max_depth = 2 for decision trees, number of hidden_layer_sizes = (3,) for MLP), avoiding overly complex structures that lead to overfitting to the training set.

3.Regularization： For the support vector machine (SVM) model, the regularization strength was adjusted by setting the parameter C = 0.1 to penalize the complex model and reduce the overfitting risk; for the Gaussian Naive Bayes (NB) model, var_smoothing = 1e-9 was used to smooth the feature variance, avoiding overfitting caused by the extreme variance of individual features.

4.Early stopping： For the multilayer perceptron in the MLP-RF hybrid model, the training process was monitored in real time during the iteration (max_iter = 1000). If the cross-validation accuracy no longer improved with the increase of training epochs, the training was stopped in advance to prevent the model from overfitting the training set noise.
